# Host Innate Immune Responses to *Acinetobacter baumannii* Infection

**DOI:** 10.3389/fcimb.2020.00486

**Published:** 2020-09-14

**Authors:** Wangxue Chen

**Affiliations:** ^1^Human Health and Therapeutics (HHT) Research Center, National Research Council Canada, Ottawa, ON, Canada; ^2^Department of Biology, Brock University, St. Catharines, ON, Canada

**Keywords:** *Acinetobacter baumannii*, innate immunity, antibiotic resistance, healthcare associated infection, virulence, vaccines, therapeutics

## Abstract

*Acinetobacter baumannii* has emerged as a major threat to global public health and is one of the key human pathogens in healthcare (nosocomial and community-acquired)-associated infections. Moreover, *A. baumannii* rapidly develops resistance to multiple antibiotics and is now globally regarded as a serious multidrug resistant pathogen. There is an urgent need to develop novel vaccines and immunotherapeutics as alternatives to antibiotics for clinical management of *A. baumannii* infection. However, our knowledge of host immune responses to *A. baumannii* infection and the identification of novel therapeutic targets are significantly lacking. This review highlights the recent advances and critical gaps in our understanding how *A. baumannii* interacts with the host innate pattern-recognition receptors, induces a cascade of inflammatory cytokine and chemokine responses, and recruits innate immune effectors (such as neutrophils and macrophages) to the site of infection for effective control of the infection. Such knowledge will facilitate the identification of new targets for the design and development of effective therapeutics and vaccines to fight this emerging threat.

## Introduction

*Acinetobacter baumannii*, a Gram-negative bacterium, has emerged as a major threat to global public health. *A. baumannii* is one of the key human pathogens in healthcare (nosocomial and community-acquired)-associated infection. The infection causes multifaceted clinical manifestations, including ventilator-associated pneumonia, catheter-associated blood and urinary tract infections, septicemia, meningitis, and burn- and wound-associated skin and soft tissue infections. *A. baumannii* infection is responsible for up to 17% of all nosocomial infections, especially amongst immunocompromised individuals (Munier et al., 2019). The majority of *A. baumannii* infections affect the respiratory tract while a very high mortality rate is seen in patients that develop bacteremia and sepsis. Moreover, *A. baumannii* rapidly develops resistance to multiple antibiotics; as such, the World Health Organization has placed carbapenem-resistant *A. baumannii* on Priority 1 (critical) of the list of bacteria for which new antibiotics are urgently needed (http://www.who.int/mediacentre/news/releases/2017/bacteria-antibiotics-needed/en/). Similarly, *A. baumannii* is regarded as a serious multidrug resistant (MDR) pathogen by the US Centers for Disease Control and Prevention and other nations.

Given the increasing difficulty in treating *A. baumannii* infections, the high mortality rates associated with the infection, and the few antimicrobials in the development pipeline, there is an urgent need to develop new antimicrobials and alternative therapeutic strategies. However, there has thus far been limited progress and success in the development of new interventions against *A. baumannii*. Immunomodulators that stimulate host innate immunity have potential as stand-alone treatment or as immune adjuvants for *A. baumannii* infection since many infected patients are immunocompromised prior to infection. However, the development of such therapies requires a better understanding the host immune response to infection by *A. baumannii*.

The majority of current studies on *A. baumannii* infections have focused on the molecular epidemiology of the infections, the distribution and mechanisms of antibiotic resistance, and the optimization of antibiotic treatment (Lee et al., 2017; Wong et al., 2017; Harding et al., 2018; Li F.J. et al., 2018; Morris et al., 2019). Relatively limited data are available on the host innate immune response to *A. baumannii* and its potential impact on the development of adaptive immunity against the infection. This review attempts to summarize and discuss the key advances and current gaps in our understanding of host innate immunity against *A. baumannii* infection with an emphasis on the results from human cell culture and animal model studies. The potential implication of such advances in the development of innovative immunotherapeutics and vaccines is also discussed.

## Virulence Factors and Pathogenesis of *A. baumannii*

*A. baumannii* has developed a number of specific and non-specific virulence factors for successful colonization and infection in the host. Detailed discussion of the potential roles and modes of action of these virulence factors are beyond the scope of this review and have been comprehensively reviewed elsewhere (Mortensen and Skaar, 2012; Lee et al., 2017; Weber et al., 2017; Wong et al., 2017; Harding et al., 2018; Li F.J. et al., 2018; Morris et al., 2019). Briefly, *A. baumannii* virulence factors play critical roles in serum/complement resistance, bacterial replication, cell adhesion and cytotoxicity, biofilm formation, immune evasion, and interaction with other bacteria during a polymicrobial infection (Lee et al., 2017; Wong et al., 2017; Harding et al., 2018; Morris et al., 2019). However, unlike many other bacterial pathogens, no distinct toxins with virulence potential were identified from *A. baumannii*. Of the currently identified virulence factors, outer membrane protein A (OmpA) is one of the best characterized. OmpA is highly conserved, with molecular similarities among different clinical isolates of *A. baumannii*, and plays an important role in the pathogenesis of other Gram-negative bacterial pathogens (Wong et al., 2017; Harding et al., 2018; Morris et al., 2019). Of particular relevance to this review, OmpA interacts with host cells to induce host inflammatory and innate immune responses, although the potential of OmpA as a target for vaccines and immunotherapeutics remains inconclusive (Chen, 2015).

Several virulence factors (such as biofilm-associated protein, OmpA, and pili) have been identified for their contribution to biofilm formation and production. However, most *A. baumannii* biofilm studies were performed *in vitro* or in animal models of acute infection. As such, the overall role of biofilm in the pathogenesis of human *A. baumannii* infection remains unclear. It appears that the ability to form biofilm is associated with the survival of *A. baumannii* in the environment, its interaction with other microbes, and with sensitivity to serum and antibiotics, rather than with the establishment of an infection in the host (Chen, 2020). Indeed, it was recently reported that virulent, opaque colonies of *A. baumannii* clinical isolate AB5075 produced less biofilm *in vitro* than the avirulent, translucent colonies (Chin et al., 2018).

*A. baumannii* has also developed redundant and complex iron utilization and regulatory mechanisms (De Leseleuc et al., 2012, 2014; Wong et al., 2017; Parquet et al., 2019; Runci et al., 2019). Iron availability or lack thereof is critical for bacterial growth and host defense (nutrition immunity), respectively; iron may also play an important role in blood dissemination during *A. baumannii*-associated pneumonia (Parquet et al., 2019). *A. baumannii* also possessed a *mumR*-mediated transcriptional mechanism for surviving Mn starvation and oxidative stress (Green et al., 2020). *mumR* mutants showed reduced fitness in a murine model of *A. baumannii* pneumonia and exogenous Mn could restore *A. baumannii* susceptibility to ROS. It appeared that *mumR* contributed to the bacterial resistance to ROS through its role in regulating phenylacetate and gamma-aminobutyric acid catabolism pathways (Green et al., 2020).

As with other Gram-negative pathogenic bacteria, *A. baumannii* uses secreted proteins to facilitate environmental and host adaption (Weber et al., 2017). To date, five secretion systems (type I, II, IV, V, and VI) have been identified in *Acinetobacter* species (Weber et al., 2017). Of particular interest was the type VI secretion system (T6SS) (Weber et al., 2017). *Acinetobacter* used T6SS mainly for competing with other bacteria. T6SS has so far not been found to mediate eukaryotic cytotoxicity, but it might play a role in antimicrobial resistance at the bacterial population level (Weber et al., 2017; Harding et al., 2018).

More recently, it has been recognized that *A. baumannii* virulence might be linked to the phenotypic switch between opaque and translucent colonies, which is under control of a TetR-type transcriptional regulator *ABUW_164*5 (Chin et al., 2018). The virulent, opaque colonies had a thicker capsule and showed >1,000 times higher tissue bacterial burdens in the infected mice than the avirulent, translucent colonies (Chin et al., 2018; Ahmad et al., 2019). The opaque colonies were also resistant to reactive oxygen species (ROS), antibiotics, and antimicrobial peptides, but they produced less amounts of biofilm and outer membrane vesicles (OMVs) than the translucent colonies. However, the OMVs from the opaque colonies were more immunogenic (Ahmad et al., 2019). Importantly, human *A. baumannii* clinical isolates studied were almost exclusively of opaque phenotype (Chin et al., 2018; Ahmad et al., 2019). The opaque colonies maintained their phenotype in the infected mice where there were about 3,000-fold increases in the number of opaque colonies from mice infected with the translucent colonies (Chin et al., 2018), suggesting that the switch from translucent to opaque phenotype is a bacterial response to selective pressure within the host.

## Host Innate Immune Responses to *A. baumannii*

Many clinical and experimental studies support an important role for the host innate defense in *A. baumannii* infection. *A*. *baumannii* generally shows no threat to healthy individuals, but causes serious infections in immunocompromised individuals and intensive care patients. Experimentally, *A. baumannii* was recognized by the host immune system at a very early stage of infection (within hours), which largely determined the host fate of the infection (Harris et al., 2013; Bruhn et al., 2015). In addition, the susceptibility to *A. baumannii* infection and the severity of the infection were significantly higher in immunocompromised mice than the conventional mice (Van Faassen et al., 2007; Chen, 2015; Garcia-Patino et al., 2017; Wong et al., 2017).

*A. baumannii* infection activates the host innate immune responses through the interaction with the host pattern recognition receptors (PRRs), which leads to the production of proinflammatory cytokines and chemokines and local recruitment of macrophages and neutrophils (Garcia-Patino et al., 2017; Wong et al., 2017). However, those responses may not always be sufficiently effective to control *A. baumannii* infection, particularly when highly virulent *A. baumannii* strains are involved. In addition, *A. baumannii* may evade the host immune mechanisms to establish a successful infection.

The majority of current knowledge on the host innate response to *A. baumannii* infection is derived from mouse models of infection and from *in vitro* studies using human and murine primary cells or cell lines. These studies have identified a number of innate immune cells and proinflammatory cytokines and chemokines which are critical in the host defense against *A. baumannii* infection. However, most of the animal models used in early studies required the use of immunosuppressive drugs or mucin to enhance the infection, which is likely to have a confounding effect on the innate immune response. More significantly, the potential relevance of those experimental observations in relation to human infections remains to be established. Therefore, substantial efforts are needed in order to fully elucidate host innate anti-*A. baumannii* mechanisms.

### Pathogen Pattern Recognition Receptors

Pattern recognition receptors (PRR) are proteins mainly expressed by innate immune cells (such as macrophages, monocytes, neutrophils, dendritic cells (DCs), and epithelial cells). PRRs play a critical role in the host innate immune system by sensing and recognizing (1) pathogen-associated molecular pattern (PAMP) components (i.e., lipopolysaccharide (LPS), OMVs, capsule) and (2) damage-associated molecular patterns (DAMPs), consisting of host cell components released during infection-induced cell damage or death. Host innate immune cells recognize specific PAMPs of *A. baumannii* through their membrane-bound PRRs, such as Toll-like receptors (TLRs), and through their cytoplasmic PRRs, such as the nucleotide-binding oligomerization domain (NOD)-like receptors (NLRs).

#### Toll-Like Receptors

TLRs are the most important membrane-bound PRRs, playing a critical role in the recognition of invading pathogens by the innate immune system. To date, 11 TLRs in humans and 12 in mice have been identified. Mice do not express TLR10, but express TLRs one to nine, and have two additional paralogs (TLR11 and 12) that are not present in humans. Each TLR type recognizes a discrete set of PAMPs, and TLRs are predominantly expressed on cells that are likely to encounter microbes. As a Gram-negative bacterium, *A. baumannii* interacts with host cells mainly by engaging TLR2 and TLR4, with CD14 as co-receptor (Garcia-Patino et al., 2017; Wong et al., 2017; Morris et al., 2019). The critical role of those TLRs in host innate immunity against *A. baumannii* infection has been established from the TLR polymorphism analysis of human patients, experimental *in vitro* cell culture, and animal model studies.

TLR4 is the key receptor for host recognition of Gram-negative bacteria, including *A. baumannii*. TLR4 interacts with the lipid A moiety of LPS, the major cell wall component of Gram negative bacteria. *In vitro* culture studies with bone marrow-derived macrophages (BMDMs) and dendritic cells (BMDCs) showed that activation of the TLR4 signaling pathway is essential for initiating an innate immune response to *A. baumannii* by macrophages and DCs. Response factors include the activation of NF-κB and mitogen-activated protein kinases (MAPKs) such as p38, ERK, and JNK; optimal production of interleukin (IL)-6, IL-12, and tumor necrosis factor (TNF)-α; inducible nitric oxide synthase (iNOS) mRNA expression and NO production; as well as the killing of *A. baumannii* (Kim et al., 2013). The engagement of TLR4 and TLR2 by both whole cells and cell components of *Acinetobacter* species has been demonstrated in human monocytic THP-1 cells and in human TLR2- and TLR4-transfected HEK-293 cells (Erridge et al., 2007; Conrad et al., 2009).

Studies using mouse and rat models have confirmed the important role of TLR4 in the host innate defense against respiratory and systemic *A. baumannii* infection (Knapp et al., 2006; Lin et al., 2012; Wang et al., 2016; Peng et al., 2018; Zhang et al., 2018). TLR4-/- mice were more susceptible to intranasal *A. baumannii* infection than wild-type mice (Knapp et al., 2006), with higher tissue bacterial burdens and more persistent infection. However, Lin et al. (2012) found that TLR4-/- mice were much more resistant to a lethal intravenous inoculation with a highly virulent strain of *A. baumannii*. In that study, all wild-type mice died of the infection whereas all TLR4-/- mice survived, despite the comparable tissue bacterial burdens between the two strains of mice (Lin et al., 2012). Apparently, virulent *A. baumannii* strains shed more LPS during bacterial replication than less virulent strains, which resulted in enhanced TLR4 activation and hyperinflammatory response. Thus, the protection seen in TLR4-/- mice in the study was mainly driven by immune regulation rather than by the bacterial burdens and associated tissue damage. This hypothesis was confirmed by the successful treatment (i.e., full protection) of wild-type mice with a novel lipid A biosynthesis inhibitor, which does not inhibit growth of the bacterium but suppresses *A. baumannii* LPS-induced activation of TLR4 (Lin et al., 2012). Similarly, it was reported that signaling via another PRR (RAGE, the receptor for advanced glycation end products) is not required in a mouse model of *A. baumannii* pneumonia, in that neither inflammation nor bacterial clearance was altered in RAGE-/- mice. RAGE, a member of the Ig superfamily, consists of a very short cytoplasmic domain and a large extracellular region containing three Ig-like domains, which is capable of binding multiple PAMPs and DAMPs (Wu et al., 2011). RAGE-/- mice systemically infected with *A. baumannii* exhibit increased survival and reduced bacterial burdens in the liver and spleen (Noto et al., 2017).

These results reflect the complexity of the host innate immune response to *A. baumannii*. The host outcome is likely to be determined by the dynamics and sequential events of such interactions. Moreover, the role of TLR4 in the host defense against *A. baumannii* can be affected by the virulence of bacterial strains, the structures and abundance of LPS, the routes (intranasal vs. intravenous or intraperitoneal) of infection, the strain and sex of the mice, and the infection model (lethal vs. sublethal) (Maclean et al., 2009; Lin et al., 2012; Vinogradov et al., 2014; Pires et al., 2020). In this regard, it was recently reported that female mice were more susceptible to intranasal infection with *A. baumannii* and treatment of male mice with estradiol increased their susceptibility to infection to a magnitude similar to female mice. The high susceptibility of female mice appeared to be associated with enhanced inflammatory cytokine responses and reduced recruitment of neutrophils and macrophages at the site of infection (Pires et al., 2020). Those findings underline the potential importance of using both sexes in studying host innate immunity against *A. baumannii* infection. However, studies by others showed no significant differences in the susceptibility between male and female mice (Breslow et al., 2011a; Harris et al., 2013).

TLR2 senses bacterial lipoproteins, peptidoglycan, and mycobacterial lipoarabinomannan. Compared to TLR4, the role of TLR2 in the host innate immune response to *A. baumannii* is less well-defined. The initial study by Knapp et al. (2006) has shown that TLR2-/- mice were more resistant to intranasal *A. baumannii* infection than wild-type mice. In that study, TLR2-/- mice showed an accelerated elimination of bacteria from the lung, which was accompanied by earlier pulmonary recruitment of inflammatory cells and increased levels of the chemokines macrophage inflammatory protein (MIP)-2 and monocyte chemoattractant protein (MCP)-1. This suggests that TLR2 signaling may inhibit the innate responses during acute *A. baumannii* pneumonia (Knapp et al., 2006). However, subsequent studies by Kim et al. showed that TLR2-/- mice were more susceptible to *A. baumannii* infection than wild-type mice, with higher lung bacterial burdens and increased levels of IL-6 and MIP-2 in the bronchoalveolar lavage (BAL) fluids (Kim et al., 2014). However, the comparable changes in body weight and lung tissue histopathology between wild-type and TLR2-/- mice suggest that TLR2 signaling plays only a marginal role in the host defense against *A. baumannii*.

The involvement of TLR2 in the host recognition of *A. baumannii* was also studied *in vitro* in human alveolar epithelial cell culture (A549) and human primary airway epithelial cells (March et al., 2010). This study showed that *A. baumannii* engaged TLR2 to activate the expression of IL-8 via NF-κB and MAPK p38- and p44/42-dependent pathways. Moreover, a sublethal concentration of recombinant OmpA stimulated substantial surface expression of TLR2 in human laryngeal epithelial HEp-2 cells and activated JNK and MAPKs to induce the production of iNOS (Kim et al., 2008). In contrast, it was reported that absence of TLR2 showed no effect on *A. baumannii*-induced cytokines production in BMDMs (Lin et al., 2012).

Collectively, it appears that *A. baumannii* interacts with the host cells via both TLR4 and TLR2 to initiate the innate immune responses. In this regard, TLR4 is largely and perhaps exclusively engaged by LPS whereas TLR2 and other TLRs can be activated by other bacterial components, including LPS. LPS-deficient *A. baumannii* was able to stimulate mouse macrophages via a TLR2-dependent mechanism and induce weak NF-κB activation and TNF-α secretion (Moffatt et al., 2013). Interestingly, the ability of *A. baumannii* LPS to stimulate TLR2 was reported to be related to the MDR (multi-drug resistance) status of the bacterial strains (Ubagai et al., 2015). However, others reported that pure LPS isolated from all *Acinetobacter* strains studied signaled exclusively via TLR4 (Erridge et al., 2007), although ultraviolet-killed *Acinetobacter* whole cells stimulated both TLR2 and TLR4 signaling. The reasons for the observed discrepancy is not clear but can be related to the differences in the LPS purity, the bacterial strains and the cells used in the studies. In addition, the pulmonary proinflammatory responses induced by intranasal inoculation of mice with *A. baumannii* OMVs were partially inhibited in TLR2-/- and TLR4-/- mice as compared to those of wild-type mice, further highlighting the important roles of those TLRs in *A. baumannii*-induced pulmonary inflammation *in vivo* (Marion et al., 2019).

Recent TLR polymorphism studies in human patients support an important role of TLRs in the host resistance to *A. baumannii* infection. In a case-control study with 423 *A. baumannii*-infected Chinese patients and 385 exposed controls, it was found that SNP of TLR-9, rs187084, and the haplotype GCG of TLR4 were significantly associated with *A. baumannii* infection after age adjustment (He et al., 2016; Chatzi et al., 2018). Similarly, a prospective Greece study on intensive care unit (ICU)-acquired infections and their clinical outcomes found an association of TLR4 (D299G and/or T399I) and TLR9-T1237C polymorphisms with increased susceptibility to specific ICU-acquired infections, such as MDR *A. baumannii*. TLR4 polymorphisms were also associated with prolonged ICU stay (Chatzi et al., 2018). These results support the notion that TLR4 and TLR9 may contribute to the host innate immunity to *A. baumannii* infection in humans.

#### Intracellular PRRs

Although an extracellular Gram-negative bacterium, *A*. *baumannii* was capable of invading mammalian epithelial cells using a zipper-like mechanism for uptake, and the process was dependent on both microfilaments and microtubules (Choi et al., 2008). *A. baumannii* cells then transiently resided and even replicated inside the host cells. The intracellular lifestyle of *A. baumannii* has also been recently confirmed in the *Drosophila* model system (Qin et al., 2020). The preliminary analysis in this model system suggested that host proteins that regulate organelle biogenesis and membrane trafficking contributed to the regulation of the intracellular lifestyle of *A. baumannii*.

To date, only a few studies have investigated the potential role of host intracellular innate immune receptors in *A*. *baumannii* infections (Choi et al., 2008; Kale et al., 2017; Dikshit et al., 2018). As discussed earlier, susceptibility to *A. baumannii* infection in human patients is linked to polymorphisms of TLR9, which is an endolysosomal PRR (He et al., 2016; Chatzi et al., 2018). The role of TLR9 in host resistance to *A. baumannii* was further investigated in a mouse model of *A. baumannii* pneumonia (Noto et al., 2015). TLR9-/- mice showed decreased proinflammatory cytokine and chemokine responses, significantly higher bacterial burdens in the lungs, increased extrapulmonary bacterial dissemination, and more severe lung pathology as compared with those in wild-type mice (Noto et al., 2015). Those results suggest that TLR9-mediated pathogen detection is important for host defense against *A. baumannii*.

Other studies have shown that the cytosolic PRRs NOD2, NOD1, and their adaptor RIP2 are important innate immune receptors that recognize intracellular *A*. *baumannii* (Bist et al., 2014; Kale et al., 2017). The NOD1/2-RIP2 axis was needed for *A. baumannii*-induced activation of NF-κB in human lung epithelial cells, but not for the activation of MAPKs pathway or the induction of optimal cytokine and chemokine responses (Bist et al., 2014). The NOD1/2 pathway contributed to the innate control of *A. baumannii* infection through the production of β-defensin 2 by airway epithelial cells (Bist et al., 2014). Gene silencing experiments in A549 cells demonstrated an important role for NOD1/2 and RIP2, resulting in increased intracellular invasion by *A. baumannii*, as well as prolonged replication and survival, in lung epithelial cells after silencing of these genes. Moreover, the role of the NOD1/2-RIP2 axis in *A. baumannii* infection is cell type specific since this axis was not involved in the interaction between human macrophages and *A. baumannii* (Bist et al., 2014). NOD2, RIP2, and other intracellular sensing receptors were also involved in the antimicrobial actions of the pyrimidine synthesis inhibitor, N-phosphonacetyl-L-aspartate, enhancing clearance of *A. baumannii* and other MDR pathogens *in vitro* as well as in *ex vivo* skin explant models (Jatana et al., 2018).

The *in vivo* role of NOD1 has not been established thus far (Kang et al., 2018) whereas NOD2 only plays a relatively moderate role in host defense against *A. baumannii in vivo* (Kale et al., 2017). Studies in a *Nod2*-/- mouse model of sublethal pulmonary infection showed that NOD2 contributes to the early, but not late, innate immunity against *A*. *baumannii* infection through enhanced ROS and reactive nitrogen species production (Kale et al., 2017). At early stages of infection, *Nod2*-/- mice demonstrated higher levels of bacterial load, neutrophil recruitment, cytokine/chemokines, and lung pathology, compared to controls. Prophylactic administration of NOD2 ligand muramyl dipeptide protected the wild-type mice from pulmonary *A*. *baumannii* challenge.

Inflammasome signaling is involved in host defenses against many microbial pathogens, but the precise mechanism by which *A. baumannii* activates inflammasomes and the roles of such signaling in host defense against *A. baumannii* infection are largely unknown. Polymorphisms in IL-1 receptor antagonist genes and inflammation-associated IL-1β levels have been implicated in MDR *A. baumannii* pneumonia in patients (Dikshit et al., 2018). *A. baumannii* infection-induced IL-1β/IL-18 production was found to be entirely dependent on the activation of NLRP3 (NOD-, LRR-, and pyrin domain-containing protein 3)-ASC-caspase-1/caspase-11 pathway. The critical role of NLRP3 inflammasomes in the host innate defense against *A. baumannii* clinical isolates was confirmed in NLRP3-/- mice, whereas the NLRP3 inflammasome was dispensable for protection against *A. baumannii* type strains (Kang et al., 2017; Dikshit et al., 2018; Li Y. et al., 2018; An and Su, 2019). These results are intriguing because most *A. baumannii* ATCC type strains are clinical isolates and they can be as virulent as many fresh clinical isolates in mice (Harris et al., 2013, 2017; Bruhn et al., 2015).

The activation of NLRP3-caspase-1-caspase-11 pathway was also involved in the *A. baumannii*-induced, TLR9-mediated release of IL-1β and IL-18 by infected lung epithelial cells (Morris et al., 2019). In addition, *A. baumannii* Omp34 activated NLRP3 inflammasomes of RAW264.7 macrophages via mitochondria-derived ROS, and led the expression of caspase-1-p10 and IL-1β in a time- and dose-dependent manner (An and Su, 2019). Moreover, activation of NLRP3 initiated macrophage pyroptosis via many different pathways, including P2X7R, K^+^ efflux, ROS production, and release of cathepsins (Kang et al., 2017). Furthermore, type I interferon (IFN) might play a critical role in epigenetic regulation of multiple cell death pathways (apoptosis, pyroptosis, and necroptosis) during *A. baumannii* infection, involving the expression of genes Zbp1, Mlkl, caspase-11, and Gsdmd as well as the activation of NLRP3 inflammasomes (Li Y. et al., 2018). Further studies are needed to dissect the relative contribution of these important innate immune pathways in the host defense against *A. baumannii* infection and the regulation of inflammatory responses.

Caspase-11-mediated innate immune responses also play a crucial role in host innate immunity against *A. baumannii*. *A. baumannii* induced the activation of both canonical and non-canonical inflammasome pathways in mouse BMDMs (Wang et al., 2017). Caspase-11-/- mice showed enhanced susceptibility, impaired bacterial clearance, and exacerbated pulmonary tissue damage following pulmonary infection with *A. baumannii* (Wang et al., 2017). Similarly, the IL-1β level in BAL fluid was impaired in *A. baumannii* infected caspase-1/11-/- mice. However, the bacterial loads in BAL fluid and lungs were comparable between wild-type and caspase-1/11-/- mice. In addition, the severity of lung pathology was reduced in NLRP3-/-, caspase-1/11-/-, and IL-1-receptor-/- mice, but the immune cell recruitment and inflammatory cytokine/chemokine production were not changed in these mice. These results suggest that *A. baumannii* activates NLRP3 inflammasome, which mediates IL-1β production and lung pathology (Kang et al., 2017). However, NLRC4 is not required for *A. baumannii*-induced production of IL-1β in macrophages and the role of caspase-11 in the clearance of *A. baumannii* remains to be determined.

### Roles of Innate Immune Cells in *A. baumannii* Recognition and Clearance

Recruitment and activation of innate immune cells to the site of infection is a hallmark of host defense against *A. baumannii* infection. The process is mediated by a coordinated proinflammatory cytokine and chemokine response. Apart from the residential macrophages, neutrophils are the first to arrive and are the most effective phagocytic cell for controlling *A. baumannii* infection. Other innate immune cells, such as macrophages, natural killer (NK) cells, and DCs, play minor to moderate roles in *A. baumannii* clearance, but secrete cytokines and chemokines that are crucial for the initiation of an effective host defense. In addition, epithelial cells release both cytokines and chemokines as well as antimicrobial peptides such as β-defensins, IL-8, and chemokine (C-X-C motif) ligand 1 (CXCL1), which assist the recruitment of neutrophils and macrophages and inhibit the growth of invading *A. baumannii*. Although substantial efforts have been made to characterize the role of neutrophils, macrophages and DCs in the host innate immunity against *A. baumannii*, a comprehensive understanding of the relative contribution of these cells and their interaction is still lacking.

#### Neutrophils

Neutrophils are the key and most potent innate immune effector that has been identified thus far in control of *A. baumannii* infection. Neutrophils control and eliminate *A. baumannii* through the release of ROS, myeloperoxidase, and β-defensins as well as the formation of neutrophil extracellular traps (NETs) (Wong et al., 2017; Morris et al., 2019). Experimental studies in the mouse and rat models of *A. baumannii* infection have shown that *A. baumannii* induces rapid expression and production of several neutrophil chemotactic cytokines and chemokines, such as MIP-2, CXCL1, and chemokine (C-C motif) ligand 5 (CCL5) (Van Faassen et al., 2007; Breslow et al., 2011a). In addition, phenylacetate from *A. baumannii* was recently identified as a pathogen-derived neutrophil chemoattractant (Bhuiyan et al., 2016).

Sublethal infection of mice with *A. baumannii* induced the rapid recruitment of large numbers of neutrophils into the site of infection (Joly-Guillou et al., 1997; Van Faassen et al., 2007; Breslow et al., 2011a); neutrophil levels then slowly decreased along with the subsequent reduction of tissue bacterial burden and eventual clearance of the infection (Van Faassen et al., 2007). Depletion of neutrophils converted an otherwise sublethal infection into a lethal one in mice and rats (Van Faassen et al., 2007; Breslow et al., 2011a; Bruhn et al., 2015). Induction of local recruitment of neutrophils by the treatment with chemotactic cytokine MIP-2 or immunomodulator 3′-5′-cyclic dimeric guanosine monophosphate enhanced the elimination of *A. baumannii* from treated mice (Van Faassen et al., 2007; Qiu et al., 2009a; Zhao et al., 2011).

The importance of neutrophils in innate immunity against *A. baumannii* infection has also been demonstrated in the mouse models of intraperitoneal or intravenous infection, and in the wound model (Breslow et al., 2011a; Tsuchiya et al., 2012; Bruhn et al., 2015; Grguric-Smith et al., 2015). The critical role of neutrophils has been established in different mouse strains including immunocompromised mice such as A/J mice, Fus1-/- mice, and diabetic mice, with *A. baumannii* strains of different sources and virulence, including hypervirulent strains (Russo et al., 2008; Luo et al., 2012; Harris et al., 2013; Hood et al., 2013; Bruhn et al., 2015). Among all the innate immune cells characterized to date, neutrophils appeared to be the most potent effector in the control of *A. baumannii* infection. Those results convincingly demonstrate a crucial role for neutrophils in host defense against both mucosal and systemic *A. baumannii* infection by limiting the local bacterial replication and subsequent systemic dissemination.

The mechanism by which the neutrophil inhibits or kills *A. baumannii* is not fully elucidated. NADPH phagocyte oxidase, but not iNOS, appears to play a critical role (Qiu et al., 2009b). In this regard, the susceptibility of gp91^phox−/−^ mice to *A. baumannii* infection was much higher than neutropenic mice (Van Faassen et al., 2007; Qiu et al., 2009b), suggesting that other innate immune cells, in addition to neutrophils, also play a role in host resistance to *A. baumannii* infection. The role of ROS in neutrophil-mediated host defense against *A. baumannii* was further supported by the experimental work in the mouse model of alcohol administration (Gandhi et al., 2014). It was shown that even physiological levels of alcohol consumption enhanced the severity of *A. baumannii* pneumonia and promoted the development of bacteraemia and sepsis. Although alcohol consumption had no direct effect on neutrophil recruitment to the lungs in uninfected mice, their leucocytes showed significantly reduced ROS production, phagocytosis, and bactericidal capacity, which leads to increased bacterial burden and severity of infection. Those results suggest that it is not only the number but also the quality and function of neutrophils that are important in host immunity against *A. baumannii* (Harris et al., 2019a).

Neutrophils may also contribute to host innate immunity against *A. baumannii* by forming NETs (Kamoshida et al., 2015, 2018; Konstantinidis et al., 2016; Lazaro-Diez et al., 2017). NETs are extracellular webs of chromatin, microbicidal proteins, and oxidant enzymes that are released by neutrophils to trap, inactivate, and kill bacteria (Papayannopoulos, 2018). *In vitro* co-culture studies have shown that human neutrophils rapidly and continuously engulfed and killed *Acinetobacter* in the first a few hours of infection, and they started to release NETs against *Acinetobacter* 3 h after infection (Lazaro-Diez et al., 2017). More importantly, it appears that macrolide antibiotics (such as clarithromycin) exerted their immunomodulatory role in *A. baumannii* infection by promoting NET formation (Konstantinidis et al., 2016). In this regard, clarithromycin could utilize autophagy to form LL-37-bearing NETs *ex vivo* and *in vivo* to inhibit *A. baumannii* growth and biofilm formation (Konstantinidis et al., 2016).

Some pathogenic *A. baumannii* strains have developed strategies to inhibit NET formation to escape host innate immune responses. One of the strategies is achieved through the inhibition of the CD11a expression on the surface of neutrophils, thereby preventing neutrophil adhesion by the pathogen (Kamoshida et al., 2015, 2018). Heat- or formalin-killed *A. baumannii* cells lost this inhibitory effect *in vitro*, and other *Acinetobacter* species did not possess it, thereby demonstrating that the observed inhibition was an active process mediated specifically by live *A. baumannii* cells (Kamoshida et al., 2018). However, further studies on the role of NETs in the control of clinical *A. baumannii* infections will be needed.

Like many other pathogenic bacteria *A. baumannii* clinical isolates could escape host innate immunity by degrading secretory IgA, a critical mucosal immune component, to enhance its colonization in the gastrointestinal tract (Ketter et al., 2018). The degradation was mediated by bacterial thioredoxin A (TrxA), a secreted bacterial reductase enzyme. *Ex vivo* treatment of excised mouse intestine sections with TrxA inhibitors significantly decreased *A. baumannii* attachment to the epithelial surface and a *trxA* targeted deletion mutant (Δ*trxA*) showed reduced gastrointestinal bacterial burdens 24 h after oral challenge (Ketter et al., 2018). *A. baumannii* also use other strategies to evade the host innate defense such as recruiting Factor H on their surface (Kim et al., 2009; King et al., 2009) and the structural similarity of their surface-associated glycans with eukaryotic sialic acids (Andolina et al., 2018).

Taken together, the experimental studies discussed above have unequivocally demonstrated a critical role for neutrophils in the innate immunity against *A. baumannii* infection; however, the precise contribution of neutrophils in clinical settings needs further investigation. Infection with *A. baumannii* is well-recognized in immunocompromised patients and is reported to be frequently isolated from neutropenic febrile patients in nosocomial settings (Karim et al., 1991), but a direct association with patients with neutropenia or neutrophil dysfunction has not been established. Limited *in vitro* studies with human neutrophils suggest that, compared to *Pseudomonas aeruginosa, A. baumannii* induces weaker responses of myeloperoxidase, ROS, and superoxide (Kamoshida et al., 2015). However, the relative efficiency of human neutrophils to kill *A. baumannii in vitro* remains controversial (Gandhi et al., 2014; Kamoshida et al., 2015) and requires further studies.

#### Macrophages

Macrophages are the other front line of innate immune cells, playing important roles in early host defense against *A. baumannii* infection by releasing chemokines for local recruitment of other innate effector cells such as neutrophils, and by killing bacteria independently and synergistically with complement. Alveolar macrophages are critical in the host control of many different respiratory bacterial pathogens. Our current knowledge on the interaction between macrophages and *A. baumannii* were largely derived from *in vitro* studies in different macrophage-like cell lines, murine BMDMs, human peripheral mononuclear cells as well as human and murine primary macrophages. Those *in vitro* studies consistently showed that both human and murine macrophages produced large amounts of proinflammatory cytokines and chemokines, and moderate amounts of iNOS, in response to *A. baumannii* infection (Breslow et al., 2011b; Qiu et al., 2012; Tsuchiya et al., 2012; Jun et al., 2013; Kim et al., 2013; Bist et al., 2014; Bruhn et al., 2015; Li et al., 2015; Kang et al., 2017, 2018, 2020; An and Su, 2019; Choby et al., 2019; Harris et al., 2019a,b; Sato et al., 2019; Lee et al., 2020). In addition, the *in vitro* uptake of *A. baumannii* by macrophages was microfilament- and microtubule-dependent (Qiu et al., 2012). As discussed above, macrophage interaction with *A. baumannii* was TLR4-dependent (Kim et al., 2013).

The importance of macrophages in host defense against *A. baumannii* infection has been confirmed in mouse and rat models of *A. baumannii* infection (Breslow et al., 2011b; Qiu et al., 2012; Tsuchiya et al., 2012; Bruhn et al., 2015; Harris et al., 2019a; Kang et al., 2020; Lee et al., 2020). Macrophages phagocytose and kill *A. baumannii* at the early stage of the infection before large numbers of neutrophils are recruited to the site of infection (Qiu et al., 2012; Bruhn et al., 2015). In addition, macrophages release critical proinflammatory cytokines and chemokines for the subsequent recruitment of neutrophils and other innate and immune cells. Depletion of macrophages in mice or rats significantly increased their susceptibility to intranasal or intravenous infection with *A. baumannii* strains of different virulence (Breslow et al., 2011b; Qiu et al., 2012; Bruhn et al., 2015; Kang et al., 2020). However, macrophages were less effective in killing *A. baumannii* than neutrophils in mice, even though large numbers of *A. baumannii* were taken up by macrophages in those studies (Qiu et al., 2012; Harris et al., 2019b).

The role of macrophages in host defense against intranasal *A. baumannii* infection was further investigated recently in C57BL/6 mice (Lee et al., 2020). Depletion of macrophages by intranasal administration of clodronate liposomes resulted in significant increases in lung bacterial burdens, enhanced local neutrophil recruitment and ROS production, associated with extensive tissue damage and systemic bacterial dissemination. However, it was surprised to note that the local levels of proinflammatory cytokines were reduced in those mice (Lee et al., 2020). The phagocytosis and bacterial killing by macrophages are at least partially dependent of the presence of IL-10 (Kang et al., 2020) in that intranasal adoptive transfer of macrophages from wild-type mice significant increased the survival and bacterial clearance of *A. baumannii-*infected IL-10-/- mice.

Taken together, those results indicate that macrophages play an important role in early host defense against *A. baumannii* infection by phagocytosis and killing of *A. baumannii* and secretion of proinflammatory cytokines and chemokines, which in turn rapidly recruit neutrophils and other innate immune cells into the site of infection. However, unlike neutrophils, macrophages are not the key immune effector in the control of *A. baumannii* infection.

#### Other Innate Immune Cells

Other innate immune cells, such as NKs, DCs, and mast cells also contribute to the innate immunity to *A. baumannii* infection, but their role remains largely inconclusive (Wong et al., 2017; Harding et al., 2018; Morris et al., 2019). NK cells play important roles in host defense against viral infections and cancers. Their role in resistance to Gram-negative bacterial pathogens including *A. baumannii* is less well-established. NK1.1 cells are recruited to the site of *A. baumannii* infection, but with delayed kinetics as compared to macrophages and neutrophils (Tsuchiya et al., 2012). NK1.1 cells recruit neutrophils at the early stage of *A. baumannii* infection by increasing CXCL1/IL-8 expression (Tsuchiya et al., 2012). The chemokine and neutrophil responses were impaired in NK1.1 cell-depleted mice and 50% of the mice died within 1 day of infection. However, multiple redundant neutrophil chemokines (such as MIP-2 and CCL5) were induced during *A. baumannii* infection (Van Faassen et al., 2007; Harris et al., 2013, 2019a,b) so it is somewhat surprising to see such a dramatic effect on neutrophil recruitment and mortality by NK1.1 cell depletion. Others have suggested that the increased number and cytotoxic activity of NK cells seen in the mouse model of intraperitoneal infection with MDR *A. baumannii* might contribute to the efficacy of the combination therapy with colistin and teicoplanin/daptomycin (Cirioni et al., 2016). However, the precise role of NK cells in host innate defense against *A. baumannii* remains to be determined.

Dendritic cells are professional antigen-presenting cells and the master regulators of the immune response by linking the non-specific innate immunity to specific adaptive immune response (Mellman, 2013). Therefore, it is not surprising that *A. baumannii* and its components (such as LPS and OmpA) interact with and activate DCs to induce and promote innate and adaptive immune responses. However, almost all the studies on the interaction between DCs and *Acinetobacter* were *in vitro* culture experiments and carried out in the early days of *A. baumannii* research. Those studies have demonstrated that OmpA induces DC maturation and Th1 immune responses in a dose-dependent manner. Low dose (5 nM) of OmpA enhanced the surface expression of CD40, CD54, CD80, and CD86 and major histocompatibility complex class I and II and the production of Th1 cytokine IL-12 (Lee et al., 2007). High dose (80 nM) of OmpA induced early-onset apoptosis and delayed-onset necrosis in DCs (Lee et al., 2010). TLR4, but not TLR2, was critical for the optimal production of IL-6, TNF-α, and IL-12 in BMDCs in response to *A. baumannii* (Kim et al., 2013). It appears that OmpA induced stronger effects on DC maturation and Th1 T cell responses than the equal amount of LPS did.

The ability of OmpA to activate DCs was further supported by the observation that OmpA might function as an adjuvant in DC-based cancer immunotherapy (Kim et al., 2011, 2012), in that OmpA-pulsed DCs significantly enhanced the number of IL-2 producing CD8^+^ T cells and IFN-γ producing CD4^+^ T cells, inhibited the tumor growth, and improved the survival of tumor-bearing mice (Lee et al., 2008; Kim et al., 2012). Although most studies showed that the immune responses induced by *A. baumannii-*activated DCs were primarily Th1 biased (Debarry et al., 2007, 2010), recent studies showed that OMVs from an *A. baumannii* clinical strain activated BMDCs to promote Th2 responses (Cai et al., 2019).

The potential role of DCs in host defense against *A. baumannii* infection *in vivo* was only reported in one study, which found that depletion of plasmacytoid DCs in mice showed no effect on their susceptibility to *A. baumannii* lung infection as compared to control IgG-treated mice (Van Faassen et al., 2007). Plasmacytoid DCs are an important DC subset that play an important role in innate immunity against low respiratory tract infections.

Mast cells play an important role in host innate immune system as sentinels for microbial pathogens and as regulatory cells in acute inflammation. The strategic location of mast cells in the epithelial and mucosal surface enabled these cells to detect invading pathogens and release cytokines and mediators to assist in early pathogen clearance (St. John and Abraham, 2013). To date, little is known on the interaction between mast cells and *A. baumannii*. It was reported that co-culture of human LAD2 mast cells with live *A. baumannii* or *P. aeruginosa* PAO1 in the presence of serum induced TNF-α and IL-8 mRNA expression, as well as cytokine production in the culture supernatant. *A. baumannii* was found to be tightly attached to the surface of LAD2 cells, suggesting a possible interaction between bacterial cells and FcγRII (CD32) on LAD2 cells. It was postulated that mast cells recognize and initiate immune responses to *A. baumannii* by releasing the pre-formed mediator TNF-α to activate neutrophils (Kikuchi-Ueda et al., 2017). However, to our best knowledge the *in vivo* role of mast cells in *A. baumannii* infection has not been reported.

Human mucosal epithelial cells (such as airway and lung epithelial cells) are critical for host defense by sensing *Acinetobacter* infection, thereby leading to the production of host defense mediators (such as IL-8 and β-defensins) to recruit neutrophils and macrophages to clear infection (March et al., 2010). Up to 58% of clinical *A. baumannii* isolates were able to attach to human lung epithelial cells (A549) with 14.5% of them being cytotoxic to the cells (Alcantar-Curiel et al., 2019). The invasion of *A. baumannii* into epithelial cells depended on a zipper-like mechanism with the involvement of microfilament and microtubule (Choi et al., 2008). *A. baumannii* induced high levels of IL-8 expression by human airway epithelial cells via NF-κB and MAPK p38 and p44/42-dependent pathways (March et al., 2010). Recent studies have also shown that *A. baumannii* infection of A549 cells induced the activation of transcription factor EB, which was required for the bacterial invasion and persistence inside the cells (Parra-Millan et al., 2018).

*A. baumannii* infection also induced the expression of human β-defensins (hBDs)-3 and, to a less magnitude, hBD-2 by human primary oral and skin epithelial cells (Feng et al., 2014). The induction was dependent on epidermal growth factor receptor signaling and the activation of MAPKs, as well as the presence of ADAM17 (a Disintegrin and Metalloprotease 17). hBDs are epithelial cell-derived cationic antimicrobial peptides that bridge the host innate and adaptive immune system (Feng et al., 2014). *A. baumannii* was susceptible to extremely low concentrations of hBD-2 and−3 *in vitro* but the *in vivo* role of hBDs in anti-*A. baumannii* is unknown. A deeper understanding of the role of epithelial cells in the early stages of *A. baumannii* infection could reveal critical determinants in infection susceptibility. In this regard, a recent review detailed the innate immune responses to *A. baumannii* infection in airways and the potential role of antimicrobial peptides in host defense against the infection (Pires and Parker, 2019).

### Complement System

As an important effector mechanism of innate immunity, the complement system is capable of killing pathogenic bacteria by activating its three separate pathways. A series of complement proteins interact in a carefully regulated cascade that eventually leads to the killing of the bacteria by either lysis or opsonisation. The role of the complement system in *A. baumannii* infection is perhaps one of the earliest characterized among the innate immune response, but their precise role in vaccine-induced acquired immunity is less consistent (Chen et al., 2010; Mortensen and Skaar, 2012).

Clinical isolates of *A. baumannii* can generally be grouped into complement-resistant (serum resistant) and complement-sensitive (serum sensitive) strains (Garcia et al., 2000; King et al., 2009). Several groups have investigated the serum resistance of clinical isolates (Kim et al., 2009; King et al., 2009; Bruhn et al., 2015) and found that the majority of the *A. baumannii* isolates were serum resistant (Kim et al., 2009; King et al., 2009; Bruhn et al., 2015). In a recent study of 252 clinical isolates associated with 52% of mortality in patients, 92% of the isolates showed varying degrees of serum resistance (Alcantar-Curiel et al., 2019).

All serum sensitive *A. baumannii* isolates were positive for C3 deposition and the alternative complement pathway was likely to be responsible for the direct killing of those *A. baumannii* strains in normal human serum (NHS) (King et al., 2009). On the other hand, there may be several potential mechanisms behind the serum resistance of *A. baumannii*. It has been suggested that serum-resistant *A. baumannii* isolates can regulate the complement pathway, especially at the primary steps by either avoiding complement C3 deposition or by inhibiting the convertase responsible for C3 cleavage and activation of the downstream cascade (King et al., 2009). As such, resistant strains accumulated little to no C3 on their cell surface when exposed to human serum (King et al., 2009). Other regulators (such as LPS, OMPs) could inhibit the activation of the complement alternative pathway. Among them, OmpA is the main complement regulator-acquiring molecule to overcome complement attack (Garcia et al., 2000). Interestingly, by screening random transposon mutant libraries, phospholipase D and penicillin-binding protein 7/8 have been attributed to the resistance of *A. baumannii* to serum and complement-mediated killing (Russo et al., 2009; Jacobs et al., 2010) although their mechanisms remain to be determined.

*A. baumannii* could also evade the complement system by the acquisition of the alternative complement pathway inhibitor factor H to their surface, which may contribute to the persistence and dissemination of *A. baumannii* in the host (Kim et al., 2009). Factor H was found on the surface of NHS-treated live *A. baumannii* in one study (Kim et al., 2009), however King et al. (2009) found that none of their 7 clinical isolates (4 sensitive and 3 resistant) recruited factor H on the surface (King et al., 2009). Furthermore, factor H was found to interact with several OMPs and, compared to the parent strain, isogenic OmpA mutant was highly susceptible to NHS, suggesting that *A. baumannii* is able to evade complement attack through the acquisition of factor H to their surface.

The *in vivo* role of complement in the host innate defense against *A. baumannii* is also inconclusive and is perhaps dependent of the *A. baumannii* strains and the route of infection, in that treatment with cobra venom factor to deplete C3 and C5 showed no effect on mice infected intranasally with an ATCC strain but significantly increased its susceptibility in the mice infected intravenously with clinical *A. baumannii* isolates of high virulence (Qiu et al., 2009a; Bruhn et al., 2015).

### Proinflammatory Cytokines and Chemokines

The induction of proinflammatory cytokine and chemokine responses by *A. baumannii* and its components (such as LPS and OMPs) is well-established *in vitro* and *in vivo*. In several studies, clinical isolates and/or their LPS were able to induce high level of TNF-α production in RAW264.7 macrophages (Rosales-Reyes et al., 2017) and TNF-α, IL-6, IL-1β, ROS/RON responses in A549 cells (Garcia et al., 1999; Alcantar-Curiel et al., 2019). However, great variations were observed in the cytokine responses to purified *A. baumannii* LPS in human umbilical cord blood mononuclear cells from 10 newborns of healthy volunteer mothers (Reuschel et al., 2019), suggesting a strong individual heterogeneity in LPS-induced inflammatory responses.

Efficient and timely recruitment of inflammatory and innate immune cells in *A. baumannii* infection is dependent on local production of multiple proinflammatory cytokines and chemokines. A diverse array of cytokines and chemokines (such as IL-1α, IL-1β, IL-6, MIP-2, CCL5, IL-8, TNF-α, IL-10, CXCL1, MCP-1, and IL-17) were consistently elevated locally or/and systemically following *A. baumannii* infection in many studies from different laboratories (Van Faassen et al., 2007; Harris et al., 2013, 2019a,b; Garcia-Patino et al., 2017; Wong et al., 2017). Other cytokines and chemokines have also been detected but with considerable variation between different studies.

Most current cytokine and chemokine response data in *A. baumannii* infection were from *in vitro* or laboratory rodent (mainly mice) studies. Their response profiles are likely affected by the experimental systems, such as *in vitro* vs. *in vivo*, the bacterial strains, and perhaps the routes of infection. More recently, it was reported that the cytokine responses to *A. baumannii* infection were also influenced by the sex of animals used (Pires et al., 2020). In addition, the relative contribution of different *A. baumannii* components (such as LPS, OMPs, and OMVs) to the cytokine and chemokine responses during the infection remain to be defined (Jun et al., 2013). The cytokine response as a biomarker for disease pathogenesis and host immunity has been attempted by several groups. For example, a significant correlation between mortality and lung levels of MIP-2, but not TNF-α, was found in mice infected intratracheally with different clinical isolates (Eveillard et al., 2010), but not in other studies (Qiu et al., 2009a; Harris et al., 2013, 2019a).

Several studies have attempted to determine the *in vivo* functionality of MIP-2, CXCL1, IFN-γ, IL-10, and IL-17 during *A. baumannii* infection. Intranasal administration of recombinant murine MIP-2 prior to *A. baumannii* infection enhanced host resistance to the infection in both immunocompromised A/J mice and immunocompetent C57BL/6 and BALB/c mice (Van Faassen et al., 2007; Qiu et al., 2009a). The increased resistance appeared associated with a rapid recruitment of large numbers of neutrophils into the site of infection without significant effect on the number of macrophages (Van Faassen et al., 2007; Qiu et al., 2009a). On the other hand, neutralization of endogenous IFN-γ or TNF-α by monoclonal antibodies showed no effect on the tissue bacterial burdens or inflammatory responses in *A. baumannii-*infected mice (Van Faassen et al., 2007). This result was perhaps not entirely surprising since later studies have shown that IFN-γ was not consistently elevated after *A. baumannii* infection (Van Faassen et al., 2007; Qiu et al., 2009a,b, 2012, 2016; Harris et al., 2013, 2019a,b). Similarly, neutralization of endogenous CXCL1 had no effect on intraperitoneal *A. baumannii* infection in mice (Breslow et al., 2011a), despite the fact that CXCL1 is a key chemotactic factor for neutrophil recruitment and was consistently detected locally or systemically at early stage of *A. baumannii* infection (Van Faassen et al., 2007; Qiu et al., 2009a,b, 2012, 2016; Harris et al., 2013, 2019a,b).

IL-17 is one of the cytokines that has received substantial attention for its potential role in host innate immunity to *A. baumannii* infection. IL-17 is produced abundantly in the lung and has been reported to play important roles in host defense against respiratory infection with multiple pathogens. One of the key functions of IL-17 is to recruit neutrophils into the site of infection. As discussed above, the neutrophil is crucial in host resistance to *A. baumannii* infection. It is therefore logical to hypothesize that IL-17 and Th17 cells may play an important role in host defense against *A. baumannii*. However, the accumulated data from mouse model studies failed to support this hypothesis at this stage (Breslow et al., 2011a; Yan et al., 2016). Although IL-17 and its different isoforms were detected in some *A. baumannii* studies (Breslow et al., 2011a,b; Qiu et al., 2016; Yan et al., 2016), administration of recombinant IL-17 or neutralization of endogenous IL-17 activities in mice showed no effect on their resistance (Breslow et al., 2011a; Yan et al., 2016). Moreover, IL-17A-/- and wild-type mice showed comparable susceptibility to *A. baumannii* infection (Breslow et al., 2011a).

Low levels of IL-10, IL-12p40, and IL-23 at the first day of *A. baumannii* infection have been attributed to the morbidity and mortality in neutropenic mice inoculated intratracheally with five *A. baumannii* strains and one *A. junii* strain (De Breij et al., 2012). IL-10 was also reported to be involved in vaccine-induced protection against *A. baumannii* challenge where the pro-inflammatory cytokines (TNF-α and IL-6) levels were reduced significantly and anti-inflammatory cytokine IL-10 increased in lungs and serum, thereby leading to decreased severity and slow progression of disease (Garg et al., 2016).

A defined contribution of IL-10 in host innate immunity against *A. baumannii* infection has been recently established in the mouse model of intranasal *A. baumannii* infection (Kang et al., 2020). Compared to wild-type mice, IL-10-/- mice showed increased pulmonary bacterial burdens, higher mortality, severe pathology, and excess local production of proinflammatory cytokines and chemokines (Kang et al., 2020). Intranasal administration of recombinant IL-10 or macrophages from wild-type mice rescued IL-10-/- mice from lethal infection by promoting the antibacterial function of macrophages (Kang et al., 2020). IL-10 up-regulated the expression of the macrophage receptor with collagenous structure (MARCO) through a STAT3-mediated pathway (Kang et al., 2020). Similarly, the increased survival of RAGE-/- mice infected with *A. baumannii* was associated with increased circulating IL-10 levels and improved bacterial clearance (Noto et al., 2017). On the other hand, the significantly increased resistance to *A. baumannii* pneumonia seen in Fus1-/- mice was thought to be associated with a significant IL-10 down-regulation and an early IL-17 up-regulation, which resulted in a significant local recruitment of neutrophils (Hood et al., 2013).

IL-10 also functions in host innate immunity against *A. baumannii* infection by way of its anti-inflammatory properties. Thus, a balanced kinetics and magnitude of the proinflammatory cellular response to *A. baumannii* infection is essential for host survival. Indeed, LpxO, an enzyme responsible for the 2-hydroxylation of lipid A modification and a potential *A. baumannii* virulence factor, was reported to activate the transcriptional factor CREB and induce IL-10 production, which in turn limited the production of inflammatory cytokines following *A. baumannii* infection (Bartholomew et al., 2019). The vaccine-induced immune protection in a mouse model of intranasal *A. baumannii* infection was associated with increased IL-10 levels and reduced numbers of neutrophils in lungs (Singh et al., 2017). Moreover, the therapeutic effect of combined lactoperoxidase and lactoferrin treatment on *A. baumannii* infection was attributed to a substantial elevation of IL-4 and IL-10 concentrations, which prevents infection-associated tissue damage (Mahdi et al., 2018).

Several recent studies have examined the role of IL-33 in host defense against *A. baumannii* infection. IL-33 is a member of the IL-1 superfamily and induces helper T cells, mast cells, eosinophils, and basophils to produce Th2 cytokines. It has been reported that IL-33 treatment reduced the mortality and associated inflammatory responses in rat models of *A. baumannii* sepsis and pneumonia by suppressing TLR4/NF-κB signaling and IL-8 and TNF-α levels (Peng et al., 2018). On the other hand, FilF vaccine-induced protection against *A. baumannii* pneumonia in mice was associated with reduced levels of pro-inflammatory cytokines TNF-α, IL-6, IL-33, IFN-γ, and IL-1β, tissue inflammation and pulmonary damage (Singh et al., 2016). However, it is somewhat interesting that the neutrophil infiltration in their studies was significantly reduced in the protected mice despite the fact that neutrophils are important in both innate immunity and vaccine-induced protection against *A. baumannii* infection (Van Faassen et al., 2007; Kuolee et al., 2015).

In summary, many cytokines and chemokines have been implicated in the early stage of host innate responses to *A. baumannii* infection through the crucial recruitment and activation of innate effector cells (such as neutrophils and macrophages). Their role in the pathogenesis and immunity against *A. baumannii* appears to be complex due to the multiple functions of most of the cytokines and chemokines. Indeed, Zeng et al. (2020) recently analyzed the lung transcriptome changes in response to lethal intranasal infection with *A. baumannii*. They showed that genes related to TNF, cytokine-cytokine receptor interaction, TLRs, NOD-like receptor, NF-κB, Jak-STAT, HIF-1 signaling pathways, apoptosis, and phagosome were significantly upregulated whereas those associated with PI3K-AKT signaling pathway, glycolysis/gluconeogenesis, amino acid, and fatty acid metabolism were downregulated. The study also confirmed the importance of neutrophil-mediated inflammatory responses of innate immune cells in host innate defense against this pathogen (Zeng et al., 2020). However, further analysis of the kinetics of these changes will be needed to better define their contribution to the innate immunity against *A. baumannii* infection.

## Interplay With Adaptive Immunity Against *A. baumannii*

Innate immune responses are important for the development of adaptive immunity against microbial infections including *A. baumannii*. Innate immune responses exert their influence on adaptive immunity through effective antigen presentation and production of appropriate cytokines (including IL-12, CD40L, IL-1, type I IFNs, and TNF-α) to guide the differentiation and development of appropriate T cell subsets and antibody responses. Interactions between *A. baumannii* and the host innate immune system likely govern the extent of bacterial replication and local inflammatory response milieu. However, the interplay between host innate and adaptive immunity has not received much attention so far. This is partially related to our limited understanding of the role of the adaptive immune system in host defense against *A. baumannii* infection.

*A. baumannii* is a Gram-negative, extracellular bacterial pathogen, therefore B cells and antibodies are likely to play important roles in host defense against infection by this pathogen; cumulative clinical and experimental data support this notion. Patients with *A. baumannii* infections developed high titers of serum specific IgM, IgA, and IgG responses (Islam et al., 2011) although some studies suggested that humoral immune responses might be related to high mortality rather than protection (Jeong et al., 2014). Experimentally, several groups have demonstrated that passive transfer of immune sera or different formats of specific antibodies (polyclonal, monoclonal, and single domain/chain) completely or at least partially protected naïve animals against *A. baumannii* challenge (Goel and Kapil, 2001; Mcconnell and Pachon, 2010; Russo et al., 2010; Mcconnell et al., 2011a,b; Luo et al., 2012; Garcia-Quintanilla et al., 2014).

Very little is currently known about the potential role of the T cell immune response to *A. baumannii* infection, apart from its helper cell role in the development of antibody responses. Limited studies in the mouse model of pulmonary infection found that more severe *A. baumannii* infection was associated with higher levels of Th1 inflammatory cytokine responses (IL-12 and IL-23) and lower levels of IL-10, suggesting that Th2 immune responses may be protective (De Breij et al., 2012). An important role for Th2 immune responses is also supported the observations that the titers of *A. baumannii* specific antibodies correlated with protection and that the passive protection against *A. baumannii* could be conferred by immune serum transfer (Luo et al., 2012). Nevertheless, the precise roles of Th1 vs. Th2 in host defense in this infection remain unknown.

Clinically, the majority of patients who become susceptible to *A. baumannii* are immunocompromised and their innate immune responses to the infection are likely defective, which could lead to an ineffective induction of adaptive immunity. In this regard, mice that recovered from a prior sublethal infection with *A. baumannii* were only marginally protected against reinfection, despite the presence of antigen-specific antibodies and T cell responses (Qiu et al., 2016), suggesting that the innate immune response, though effective against the acute *A. baumannii* infection, appears to have induced a relatively weak adaptive immunity.

## Conclusion and Perspective

Substantial and encouraging progress has been made over the last two decades in our knowledge on the host innate immune responses to *A. baumannii* infection. It is now recognized that the course of infection in the host is determined by innate immune effector cells and by the pathogen at the very early stage of the infection (Bruhn et al., 2015). The accumulated *in vitro* and *in vivo* experimental studies have confirmed a central and critical role of neutrophils in innate immunity against *A. baumannii* infection. Neutrophils are recruited within hours to the site of infection, and then these cells uptake and kill the pathogens with high efficiency by releasing ROS and other bactericidal substances, along with the formation of NETs. However, the significance of neutrophils in the human infection remains to be confirmed and better defined.

The collective data from polymorphism analysis in patients, *in vitro* human and murine cell culture, and animal models have confirmed TLR4 as the major PRR for the initial interaction between *A. baumannii* and host cells. However, studies also indicate that other TLRs, particularly TLR2, have divergent roles in the host interaction with *A. baumannii*. Many factors such as the route of infection as well as the stage and severity of the infection have a profound effect on the function of TLRs. In this regard, it is intriguing to note the contribution of several intracellular innate immune sensing and signaling molecules (such as TLR9 and NOD2) in the immune response to *A. baumannii*, an extracellular bacterial pathogen. Further studies on the potential importance of *A. baumannii* as an intracellular pathogen will provide better understanding of the pathogenesis of the infection and the ensuing immune response. The recent development of *Drosophila* S2 cells as a model system for studying the intracellular lifestyle of *A. baumannii* (Qin et al., 2020) should facilitate the rapid identification of host factors that may contribute to intracellular infection with *A. baumannii*.

*A. baumannii* infection induces local and systemic proinflammatory cytokine and chemokine responses, which are critical for the recruitment and activation of inflammatory and innate effector cells at the site of infection. Limited studies of mouse models suggest that endogenous IL-10 and IL-33 are important in host inflammatory responses and innate immunity to *A. baumannii* infection, whereas IL-17, TNF-α, IFN-γ, and CXCL1 do not appear to be important. Current studies on the cytokine and chemokine responses to *A. baumannii* are still largely at the profiling stage. The relative contribution of the changes in the cytokine responses to host defense, infection pathogenesis, and disease outcome remains unknown. Even less is known of the interaction between different cytokines and chemokines and their network induced by the infection. In many cases, the bacterial burden and tissue damage are the major driver of the magnitude of cytokine production (Nielsen et al., 2017), reflecting the complexity of host and pathogen interaction, the multiple functions of a given cytokine, and the redundancy of the cytokine responses.

Despite the above encouraging advances, considerable gaps remain in our knowledge of host innate immune responses to *A. baumannii* infection. Our current knowledge has been largely derived from the various *in vitro* cell culture experiments, and studies with model organisms and animal models of *A. baumannii* infection. Further studies will be needed to address the relevance of those data to human infections and the eventual translation of that knowledge into clinical applications. As with early stages of research in many other infections, better sharing of the research protocols and reagents, as well as improved reporting of experimental results (such as the animal model, cell sources, bacterial strain, and experimental protocol details), are likely to improve the data quality, experimental reproducibility, and the conclusion validation. It is highly possible that the observed differences in the innate immune responses in many studies were related to the strain, virulence, inocula dose, and routes of infection, as well as the strains of mice and the additional manipulation of the mice and pathogens.

Our knowledge on the host innate immune responses to *A. baumannii* infection remains rudimentary without a working hypothesis. Compared to many other Gram-negative bacterial pathogens, only very few innate immune components have their *in vivo* functions experimentally confirmed in *A. baumannii* infection. In addition, the potential contribution of innate lymphoid cells and different subsets of macrophages in host innate immunity to *A. baumannii* remains to be explored. Last but not least, substantial efforts will be needed to understand how innate immunity determines and shapes the development of adaptive immunity to *A. baumannii*. Such knowledge will allow for better design and development of efficient immunotherapeutics and vaccines to combat this important, emerging MDR pathogen.

## Author Contributions

The author confirms being the sole contributor of this work and has approved it for publication.

## Conflict of Interest

The author declares that the research was conducted in the absence of any commercial or financial relationships that could be construed as a potential conflict of interest.
